# Development of a biomarker panel for assessing cardiovascular risk in diabetic patients with chronic limb-threatening ischemia (CLTI): a prospective study

**DOI:** 10.1186/s12933-023-01872-x

**Published:** 2023-06-12

**Authors:** Elisabetta Nardella, Federico Biscetti, Maria Margherita Rando, Andrea Leonardo Cecchini, Maria Anna Nicolazzi, Enrica Rossini, Flavia Angelini, Roberto Iezzi, Luis H. Eraso, Paul J. Dimuzio, Dario Pitocco, Massimo Massetti, Antonio Gasbarrini, Andrea Flex

**Affiliations:** 1 Cardiovascular Internal Medicine, Fondazione Policlinico Universitario A. Gemelli IRCCS, Largo Agostino Gemelli 8, Roma, 00168 Italy; 2 Università Cattolica del Sacro Cuore, Largo Francesco Vito 1, Roma, 00168 Italy; 3 Radiology Unit, Fondazione Policlinico Universitario A. Gemelli, IRCCS, Roma, Italy; 4 Division of Vascular and Endovascular Surgery, Thomas Jefferson University, Philadelphia, PA USA; 5 Diabetology Unit, Fondazione Policlinico Universitario A. Gemelli, IRCCS, Roma, Italy; 6 Department of Cardiovascular Sciences, Fondazione Policlinico Universitario A. Gemelli IRCCS, Largo Agostino Gemelli 8, Roma, 00168 Italy; 7 Department of Medical and Surgical sciences, Fondazione Policlinico Universitario A. Gemelli IRCCS, Largo Agostino Gemelli 8, Roma, 00168 Italy

**Keywords:** Peripheral artery disease (PAD), Chronic limb threatening ischemia (CLTI), Major adverse cardiac events (MACE), Major adverse limb events (MALE), Biomarkers

## Abstract

**Background:**

Lower-extremity endovascular revascularization (LER) is often required for diabetic patients with chronic limb threatening ischemia (CLTI). During the post-revascularization period patients may unpredictably experience major adverse cardiac events (MACE) and major adverse limb events (MALE). Several families of cytokines are involved in the inflammatory process that underlies the progression of atherosclerosis. According to current evidence, we have identified a panel of possible biomarkers related with the risk of developing MACE and MALE after LER. The aim was to study the relationship between a panel of biomarkers - Interleukin-1 (IL-1) and 6 (IL-6), C-Reactive Protein (CRP), Tumor Necrosis Factor-α (TNF-α), High-Mobility Group Box-1 (HMGB-1), Osteoprotegerin (OPG), Sortilin and Omentin-1- at baseline, with cardiovascular outcomes (MACE and MALE) after LER in diabetic patients with CLTI.

**Methods:**

In this prospective non-randomized study, 264 diabetic patients with CLTI undergoing endovascular revascularization were enrolled. Serum levels of each biomarker were collected before revascularization and outcomes’ incidence was evaluated after 1, 3, 6 and 12 months.

**Results:**

During the follow-up period, 42 cases of MACE and 81 cases of MALE occurred. There was a linear association for each biomarker at baseline and incident MACE and MALE, except Omentin-1 levels that were inversely related to the presence of MACE or MALE. After adjusting for traditional cardiovascular risk factors, the association between each biomarker baseline level and outcomes remained significant in multivariable analysis. Receiver operating characteristics (ROC) models were constructed using traditional clinical and laboratory risk factors and the inclusion of biomarkers significantly improved the prediction of incident events.

**Conclusions:**

Elevated IL-1, IL-6, CRP, TNF-α, HMGB-1, OPG and Sortilin levels and low Omentin-1 levels at baseline correlate with worse vascular outcomes in diabetic patients with CLTI undergoing LER. Assessment of the inflammatory state with this panel of biomarkers may support physicians to identify a subset of patients more susceptible to the procedure failure and to develop cardiovascular adverse events after LER.

**Supplementary Information:**

The online version contains supplementary material available at 10.1186/s12933-023-01872-x.

## Background

Peripheral artery disease (PAD) is a frequent complication of type 2 diabetes mellitus (T2DM) [1]. One of its most dangerous manifestations is chronic limb threatening ischemia (CLTI), characterized by ischemic rest pain, tissue loss and/or gangrene [2]. Lower-extremity endovascular revascularization (LER) is often required to avoid limb loss and acute limb ischemia [3, 4]. However, neither LER nor open surgical revascularization guarantee short and/or long-term success in all patients [5]. Despite best clinical efforts to control risk factors, clinical outcomes after LER revascularization have a significant heterogeneity based on technique and anatomical disease location in patients with similar subset of medical comorbidities [6–8]. Furthermore, there is extensive clinical evidence of the unpredictably of major adverse cardiac events (MACE) and major adverse limb events (MALE) in diabetic and non-diabetic patients undergoing LER [6, 7, 9] suggesting the possibility of alternative mechanistic pathways as key determinants of clinical outcomes after LER beyond traditional atherosclerotic risk factors.

Scientific evidence has shown that an inflammatory process underlies the progression of atherosclerosis [10–12]. Several families of cytokines are involved in this inflammatory state [10–12]. We hypothesized that patients with an activation of the systemic inflammatory cascade and reduction of endogenous anti-inflammatory mechanisms have worse outcomes. According to current evidence, we have identified a panel of biomarkers that in a more sensitive and specific way could be correlated with the risk of developing MACE and MALE in diabetic patients with CLTI after LER. Interleukin (IL)-1 is a potent pro-inflammatory cytokine which is involved in the atherosclerotic process [13]. It has also been studied as a therapeutic target [14]. High levels of IL-6, C-Reactive Protein (CRP), Tumor Necrosis Factor (TNF)-α contribute with the plaque formation. In our previous studies, we found a relationship between them and PAD complications [7, 12, 15–20]. High-Mobility Group Box-1 (HMGB-1) plays a role in inflammation, angiogenesis and tissue regeneration [21, 22]. We recently demonstrated an association between baseline serum HMGB-1 levels with the outcomes in diabetic PAD patients with CLTI [23]. Osteoprotegerin (OPG) is involved in vascular calcification and plaque stability [18, 24, 25]. Sortilin is a protein that play a role in apolipoproteins trafficking and in the formation of foam cells [26–28]. Omentin-1 is an adipokine with anti-inflammatory properties [29]. We recently demonstrated that its levels are inversely related with the presence of PAD and worse outcomes in diabetic patients [30, 31].

The aim of this study is to assess the relationship between the baseline levels of these biomarkers and the outcomes after LER, in particular MACE and MALE, in diabetic patients with CLTI undergoing revascularization.

## Methods

### Study design

This study was approved by the Ethics Committee of the *Fondazione Policlinico Universitario A. Gemelli Istituto di Ricovero e Cura a Carattere Scientifico (IRCCS)* and adhered to the principles of the Declaration of Helsinki. All the individuals agreed to participate in the study and gave informed consent. This clinical protocol was designed as a prospective non-randomized study to verify the relationship between the serum levels of a panel of biomarkers (IL-1, IL-6, CRP, TNF-α, HMGB-1, OPG, Sortilin and Omentin-1) and the incidence of MACE and MALE after LER performed in diabetic patients with CLTI.

### Study population and clinical assessment

We evaluated consecutive patients with diabetes and CLTI admitted to the Department of Cardiovascular Internal Medicine at *Fondazione Policlinico Universitario A. Gemelli IRCCS*, Rome, Italy, from December 1, 2019 to January 31, 2022. At the recruitment visit, clinical, laboratory, ultrasound and radiological data of the patients were collected. The diagnosis of CLTI of at least one limb, category 4, 5 or 6 of PAD in accordance with the Rutherford classification [32], and the indication to LER was confirmed according to the diagnostic criteria accepted by international guidelines [33]. Exclusion criteria were inability or refusal to sign informed consent for study inclusion, age under 18, LER or previous lower limb bypass surgery within the past 3 months, diabetic peripheral neuropathy, systemic steroid use or a prior history of use in the previous month, pregnancy, renal disease with estimated glomerular filtration rate (eGFR) < 30 ml/min, active cancer, life expectancy < 12 months, known liver disease with a functional status of B or above according to the Child–Pugh classification, congenital or acquire thrombophilia, active autoimmune disease, need for ongoing oral anticoagulant therapy, anemia severe requiring blood transfusion, inability or impossibility to undergo periodic follow-up and to adhere to therapy, active acute infectious disease or in the two weeks preceding LER, diabetic foot ulcers with sign of infection or osteomyelitis, failure of the procedure. Overall, 264 were enrolled and followed for the entire duration of the study. For all patients, additional clinical data was collected, including age, body mass index (BMI), history of cardiovascular diseases (CAD), cerebrovascular disease (CVD), hypertension, hypercholesterolemia, smoking status, renal failure. For all patients, the lipid profile was evaluated.

All patients were taking lipid-lowering therapy to achieve a low-density lipoprotein cholesterol (LDL-C) target of less than 55 mg/dL.

At the time of enrollment all patients were on a single antiplatelet drug, and after LER they took dual antiplatelet therapy (aspirin and clopidogrel) for 1 month.

### Revascularization treatment and follow‑up

Balloon angioplasty and, if indicated, arterial stenting were performed according to standard techniques [34]. The procedure was considered successful if the residual arterial stenosis was less than 30% [35]. We excluded from follow-up 13 (4.69%) of 277 originally included patients due to primary treatment failure after revascularization. No major complications, defined according to the definitions of the Society of Interventional Radiology [35], were observed.

For the follow-up, patients were evaluated 1, 3, 6 and 12 months after the LER to assess incidence of MACE and MALE. MACE was defined as composite of myocardial infarction, stroke and cardiovascular death [36]. MALE was defined as composite of acute limb ischemia, major vascular amputations, limb-threatening ischemia leading to urgent revascularization [7, 36].

### Blood sampling procedures and biochemical assays

Blood sampling of patients was performed at baseline, before LER, after an overnight fast. Fasting glucose (FBG), serum creatinine, total cholesterol, LDL-C, triglycerides, and glycated hemoglobin (HbA1c), were determined. Renal function was calculated using eGFR, according to the Chronic Kidney Disease Epidemiology Collaboration (CKD-EPI) formula [37]. Serum was prepared by centrifugation of blood samples, which was stored at − 80 °C until assayed. Serum levels of IL-1, IL-6, TNF-α, CRP, HMGB-1, OPG, Sortilin and Omentin-1 levels were determined by commercially available ELISA kits (MBS012415 for IL-1, EH0201 for IL-6, EH0302 for TNF-α, EH0099 for CRP, EH0884 for HMGB-1, EH0247 for OPG, EH15347 for Sortilin, MBS167112 for Omentin-1; Aurogene) according to their protocols. The intra- and inter-assay coefficients of variation were 3.5 and 10.5%, respectively. The sensitivity, defined as the mean ± 3 SD of the 0 standard, was calculated to be 0.15 pmol/mL. For each patient, the serum levels were measured twice, and the results were averaged.

### Statistical analysis

Based on the previous data, we assumed an effect size of 0.20. To have a power (1 – β) of 90% and considering an α error of 5% (0.05), a total of at least 255 patients were required. Data were summarized as means (standard deviations, SDs) for continuous variables and counts (percentages) for categorical variables. Demographic and clinical data of the population were compared using chi-square and t-test. We assessed the association between each biomarker and the risk of developing MALE and MACE by means of logistic regression models in which the outcome of interest was entered as the dependent variable and each biomarker was entered as a continuous independent variable. We assessed whether knowledge of the biomarker values would lead to improved prognostic prediction regarding the outcomes of interest by constructing receiver operating characteristic (ROC) curves for a model including only traditional risk factors (age, sex, BMI, high blood pressure, diabetes duration, smoking status, Rutherford staging, previous cardiovascular and cerebrovascular events, treatment, total cholesterol, LDL-C, triglycerides, FBG, HbA1c) and for a model including all the aforementioned risk factors plus all biomarkers as continuous variables. We then compared the areas under the ROC curves using the roccomp function in Stata software; this function tests the equality of two ROC areas obtained by applying two or more test modalities to the same sample or to independent samples. All statistical analyses were performed separately for each of the two outcomes of interest.

Statistical significance was established at p < 0.05. All analyses were performed with Stata software version 17.0 for MacOS (Statistics/Data Analysis, Stata Corporation, College Station, TX, USA) and SPSS version 25.0 for MacOS (IBM Corporation, Armonk, NY, USA).

## Results

### Demographic and clinical characteristics

A population of 264 diabetic patients with CLTI was enrolled and followed up for the entire duration of the study. The mean age of patients was 73.4 ± 8.8 years. Of these, 171 (64.8%) were males. The median duration of T2DM was 16.2 ± 13.7 years. The mean BMI was 26.4 ± 5.0 kg/m2. Comprehensively, we included 63 (23.8%) smokers and 120 (45.5%) former smokers. Among all patients, 204 (77.3%) had arterial hypertension, 243 (92.0%) had hypercholesterolemia, 117 (44.3%) had history of CAD and 99 (37.5%) patients had history of CVD. LDL-C (71.4 ± 28.5 mg/dL) and HbA1c (7.1 ± 1.2%) were only slightly above target according to guidelines in force at the time of recruitment [38]. Taking into account the characteristics of the PAD, the Rutherford staging included 54 (20.4%) category 3 patients, 108 (40.9%) category 5 patients and 42 (15.9%) category 6 patients. The mean baseline levels of IL-1 were 77.4 ± 18.8 pg/mL, of IL6 were 56.3 ± 25.7 pg/mL, of CRP were 21.3 ± 18.0 mg/L, of TNF-α were 45.3 ± 20.8 pg/mL, of OPG were 509.9 ± 182.1 pg/mL, of HMGB-1 were 591.2 ± 197.4 pg/mL, of Omentin-1 were 28.0 ± 11.3ng/mL and of Sortilin were 1.6 ± 0.5 ng/mL. Complete clinical characteristics are shown in Table 1.

**Table 1:** Demographic characteristics of the study cohort at baseline

Number of patients	264
Men/female, n	171:93
Age, years ± SD	73.4 ± 8.8
Diabetes duration, years ± SD	16.2 ± 13.7
BMI, Kg/m^2^ ± SD	26.4 ± 5.0
Smoking (current), n (%)	63 (23.8)
Smoking (former), n (%)	120 (45.5)
Hypertension, n (%)	204 (77.3)
Hypercholesterolemia, n (%)	243 (92.0)
CAD, n (%)	117 (44.3)
CVD, n (%)	99 (37.5)
Rutherford II-4, n (%)	54 (20.4)
Rutherford III-5, n (%)	108 (40.9)
Rutherford III-6, n (%)	42 (15.9)
HbA1c, % ± SD	7.1 ± 1.2
FBG, mg/dL ± SD	132.8 ± 54.5
Total cholesterol, mg/dL ± SD	134.5 ± 34.8
LDL cholesterol, mg/dL ± SD	71.4 ± 28.5
HDL cholesterol, mg/dL ± SD	38.3 ± 11.9
Triglycerides, mg/dL ± SD	119.3 ± 63.5
Creatinine, mg/dL ± SD	1.8 ± 1.8
IL1, pg/mL ± SD	77.4 ± 18.8
IL6, pg/mL ± SD	56.3 ± 25.7
CRP, mg/L ± SD	21.3 ± 18.0
TNFα, pg/mL ± SD	45.3 ± 20.8
OPG pg/mL ± SD	509.9 ± 182.1
HMGB1, pg/mL ± SD	591.2 ± 197.4
Omentin1, ng/mL ± SD	28.0 ± 11.3
Sortilin, ng/mL ± SD	1.6 ± 0.5

Data are reported as means (standard deviation) for continuous variables and numbers (percentages) for categorical variables. BMI, body mass index; CAD, coronary artery disease; CVD, cerebrovascular disease; FBG, fasting blood glucose; eGFR, estimated glomerular filtration rate

### Biomarkers values and risk of MACE at 12 months

**Fig. 1:**
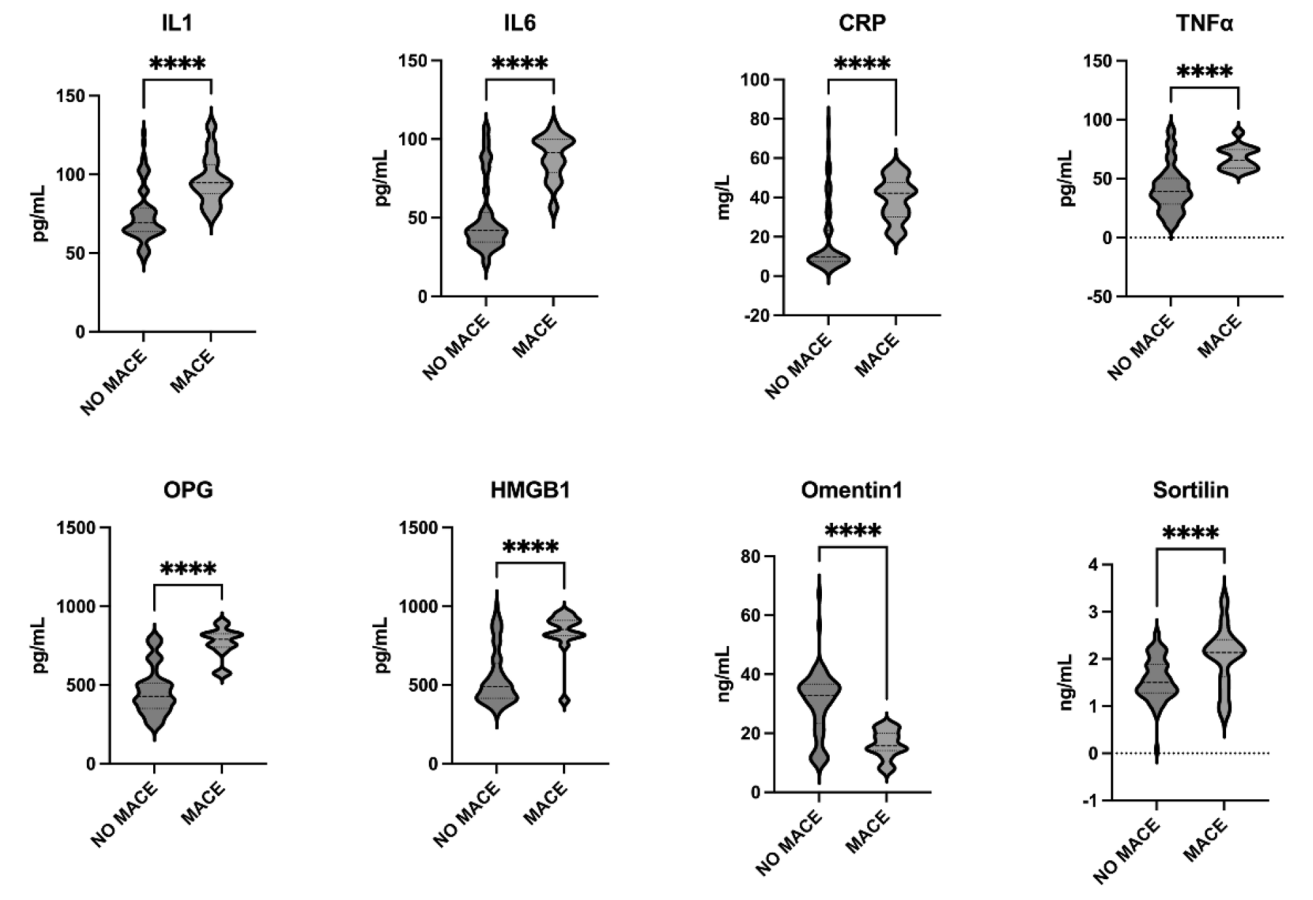
Cytokines levels in patients with and without MACE Baseline biomarkers levels according to MACE. On the violin plots, central line represents the median, upper line represents the upper interquartile range (IQR) and the lower line represents the lower IQR. **** = p < 0.0001

During the 12-month follow-up period, 42 (15.9%) patients experienced MACE after LER. Patients with MACE had diabetes for longer duration (25.6 ± 14.2 years vs. 14.4 ± 12.9 years, p < 0.01), higher BMI (29.9 ± 6.4 Kg/m^2^vs. 25.8 ± 4.4 Kg/m^2^, p < 0.01), already history of CAD (85.7% vs. 36.5%, p < 0.01) and of hypercholesterolemia (204 vs. 39 patients, p = 0.04), higher creatinine serum levels (2.7 ± 2.2 mg/dL vs. 1.6 ± 1.7 mg/dL, p < 0.01), compared to patients without MACE. There were no differences between patients with MACE and without MACE in LDL-C and HbA1c levels. Complete clinical data from patients with MACE and without MACE are shown in **Supplementary Table 1**. Considering baseline biomarkers levels, patients with MACE had higher baseline serum levels, except Omentin-1 levels that were lower in patients with MACE compared with patients without MACE (Fig. 1).

The multivariate logistic regression analysis showed that, after adjustments for the cardiovascular risk factors age, male sex, BMI, having been a smoker, hypertension, CAD, CVD, LDL-C, FBG, HbA1c, IL-6, CRP and OPG levels were independent determinant for MACE after LER in diabetic patients with CLTI (p < 0.01) (Table 2).

**Table 2:** Multivariable logistic regression for MACE

	Coef.		St.Err.	t-value		p-value	[95% Conf		Interval]	Sig
Male sex	-0.062		0.042	-1.46		0.147	-0.145		0.022	
Age	0.002		0.002	0.89		0.375	-0.002		0.006	
Diabetes duration	0.001		0.002	0.63		0.532	-0.002		0.004	
BMI	0.009		0.004	2.31		0.022	0.001		0.016	*
Smoking (current)	0.007		0.058	0.12		0.905	-0.107		0.121	
Smoking (former)	-0.142		0.045	-3.17		0.002	-0.229		-0.054	**
Hypertension	0.046		0.047	0.97		0.332	-0.047		0.139	
Hypercholesterolemia	0.215		0.070	3.05		0.003	0.076		0.354	**
CAD	0.103		0.038	2.69		0.008	0.027		0.178	**
CVD	-0.031		0.041	-0.75		0.455	-0.111		0.050	
Rutherford II-4	0.103		0.053	1.96		0.052	-0.001		0.207	
Rutherford III-5	0.166		0.048	3.50		0.001	0.072		0.260	**
Rutherford III-6	0.190		0.069	2.76		0.006	0.054		0.326	**
HbA1c	-0.078		0.023	-3.46		0.001	-0.123		-0.033	**
FBG	0.000		0.000	1.18		0.239	0.000		0.001	
Total cholesterol	0.000		0.003	0.06		0.956	-0.005		0.005	
LDL cholesterol	0.002		0.003	0.67		0.503	-0.003		0.007	
HDL cholesterol	-0.004		0.003	-1.30		0.195	-0.011		0.002	
Triglycerides	-0.001		0.001	-2.08		0.039	-0.002		0.000	*
Creatinine	0.028		0.009	3.00		0.003	0.010		0.046	**
IL1	0.001		0.001	0.91		0.362	-0.001		0.004	
IL6	0.005		0.001	3.38		0.001	0.002		0.007	**
CRP	-0.007		0.002	-3.88		0.000	-0.010		-0.003	**
TNFα	0.001		0.001	0.78		0.434	-0.002		0.004	
OPG	0.001		0.000	5.89		0.000	0.001		0.001	**
HMGB1	0.000		0.000	1.48		0.139	0.000		0.001	
Omentin1	0.001		0.002	0.41		0.679	-0.004		0.006	
Sortilin	-0.050		0.047	-1.07		0.286	-0.143		0.042	
Constant	-0.819		0.326	-2.51		0.013	-1.462		-0.175	*
Mean dependent var		0.149			SD dependent var			0.357		
R-squared		0.679			Number of obs			222.000		
F-test		14.551			Prob > F			0.000		
Akaike crit. (AIC)		-22.848			Bayesian crit. (BIC)			75.829		

** p < 0.01, * p < 0.05

### Biomarkers values and risk of MALE at 12 months

**Fig. 2:**
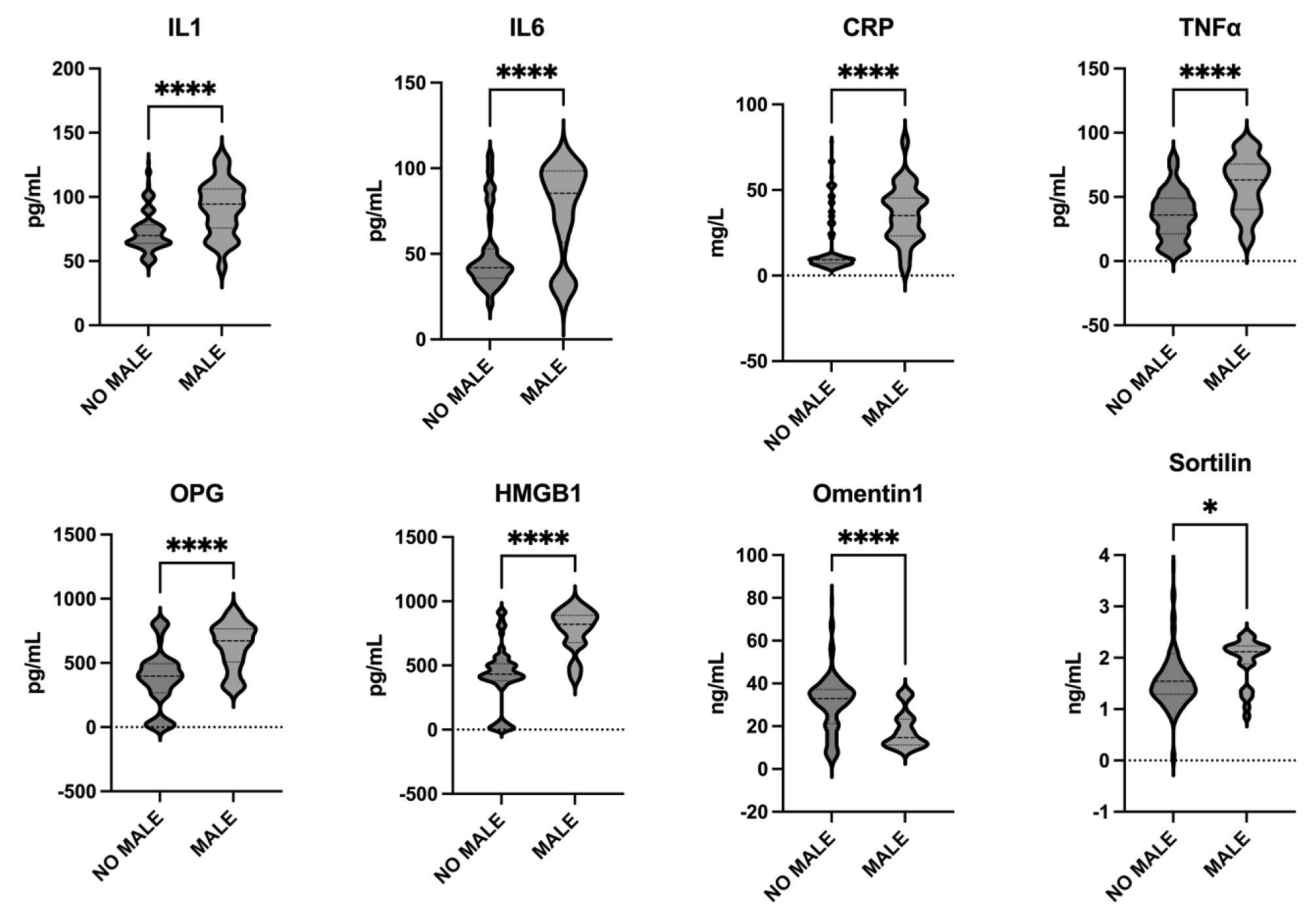
Cytokines levels in patients with and without MALE Baseline biomarkers levels according to MALE. On the violin plots, central line represents the median, upper line represents the upper interquartile range (IQR) and the lower line represents the lower IQR. **** = p < 0.0001, * = p < 0.05

During the 12-month follow-up period, 81 (30.7%) patients experienced MALE after LER. Patients with MALE were younger (p < 0.01), had more history of hypercholesterolemia (p < 0.01), of CAD (p < 0.01) and of CVD (p = 0.02), and had lower HDL-C levels than patients who did not have MALE (p < 0.01). Furthermore, Rutherford category 4 was more represented in patients undergoing MALE (p = 0.01). There were no differences between patients with MALE and without MALE in terms of diabetes duration (p = 0.67), BMI (p = 0.14), history of high blood pressure (p = 0.41), glycated hemoglobin levels (p = 0.13), LDL-C levels (p = 0.20), Rutherford category 5 (p = 0.97) and category 6 (p = 0.44), and creatinine (p = 0.59). Complete clinical data from patients with MALE and without MALE are shown in **Supplementary Table 2**. Considering baseline biomarkers levels, patients with MALE had higher baseline serum levels, except Omentin-1 levels that were lower in patients with MALE compared with patients without MALE (Fig. 2).

The multivariate logistic regression analysis showed that, after adjustments for the cardiovascular risk factors, age, Rutherford stages II-4 and III-6, HbA1c, total cholesterol, CRP, OPG, HMGB-1, and Omentin-1 were independent determinant for MALE after LER in diabetic patients with CLTI (p < 0.01) (Table 3).

**Table 3:** Multivariable logistic regression for MALE

	Coef.		St.Err.	t-value		p-value	[95% Conf		Interval]	Sig
Male sex	-0.034		0.056	-0.60		0.551	-0.145		0.078	
Age	-0.011		0.003	-3.91		0.000	-0.017		-0.006	**
Diabetes duration	0.002		0.002	0.84		0.402	-0.002		0.006	
BMI	0.005		0.005	0.94		0.347	-0.005		0.014	
Smoking (current)	0.037		0.077	0.49		0.626	-0.114		0.189	
Smoking (former)	-0.029		0.059	-0.48		0.631	-0.145		0.088	
Hypertension	-0.041		0.063	-0.65		0.517	-0.164		0.083	
Hypercholesterolemia	0.072		0.094	0.77		0.444	-0.113		0.256	
CAD	0.105		0.051	2.07		0.040	0.005		0.205	*
CVD	0.028		0.054	0.52		0.601	-0.079		0.135	
Rutherford II-4	-0.157		0.070	-2.25		0.025	-0.295		-0.020	*
Rutherford III-5	-0.021		0.063	-0.33		0.740	-0.146		0.104	
Rutherford III-6	-0.422		0.091	-4.62		0.000	-0.602		-0.242	**
HbA1c	0.097		0.030	3.25		0.001	0.038		0.157	**
FBG	-0.001		0.001	-1.61		0.108	-0.002		0.000	
Total cholesterol	-0.008		0.004	-2.35		0.020	-0.015		-0.001	*
LDL cholesterol	0.006		0.003	1.72		0.088	-0.001		0.013	
HDL cholesterol	0.005		0.004	1.20		0.232	-0.003		0.014	
Triglycerides	0.001		0.001	0.83		0.408	-0.001		0.002	
Creatinine	-0.013		0.012	-1.01		0.312	-0.037		0.012	
IL1	-0.002		0.002	-0.84		0.401	-0.005		0.002	
IL6	0.001		0.002	0.59		0.557	-0.002		0.005	
CRP	0.010		0.002	4.16		0.000	0.005		0.014	**
TNFα	0.001		0.002	0.34		0.731	-0.003		0.004	
OPG	-0.001		0.000	-5.25		0.000	-0.002		-0.001	**
HMGB1	0.001		0.000	4.22		0.000	0.000		0.001	**
Omentin1	-0.011		0.003	-3.45		0.001	-0.017		-0.005	**
Sortilin	0.055		0.062	0.88		0.381	-0.068		0.178	
Constant	1.042		0.433	2.41		0.017	0.188		1.897	*
Mean dependent var		0.270			SD dependent var			0.445		
R-squared		0.636			Number of obs			222.000		
F-test		12.034			Prob > F			0.000		
Akaike crit. (AIC)		103.365			Bayesian crit. (BIC)			202.042		

** p < 0.01, * p < 0.05

### Improvement in the Prediction of Cardiovascular Events After Adding the panel of biomarkers to Established Clinical and Laboratory Risk Factors

**Fig. 3:**
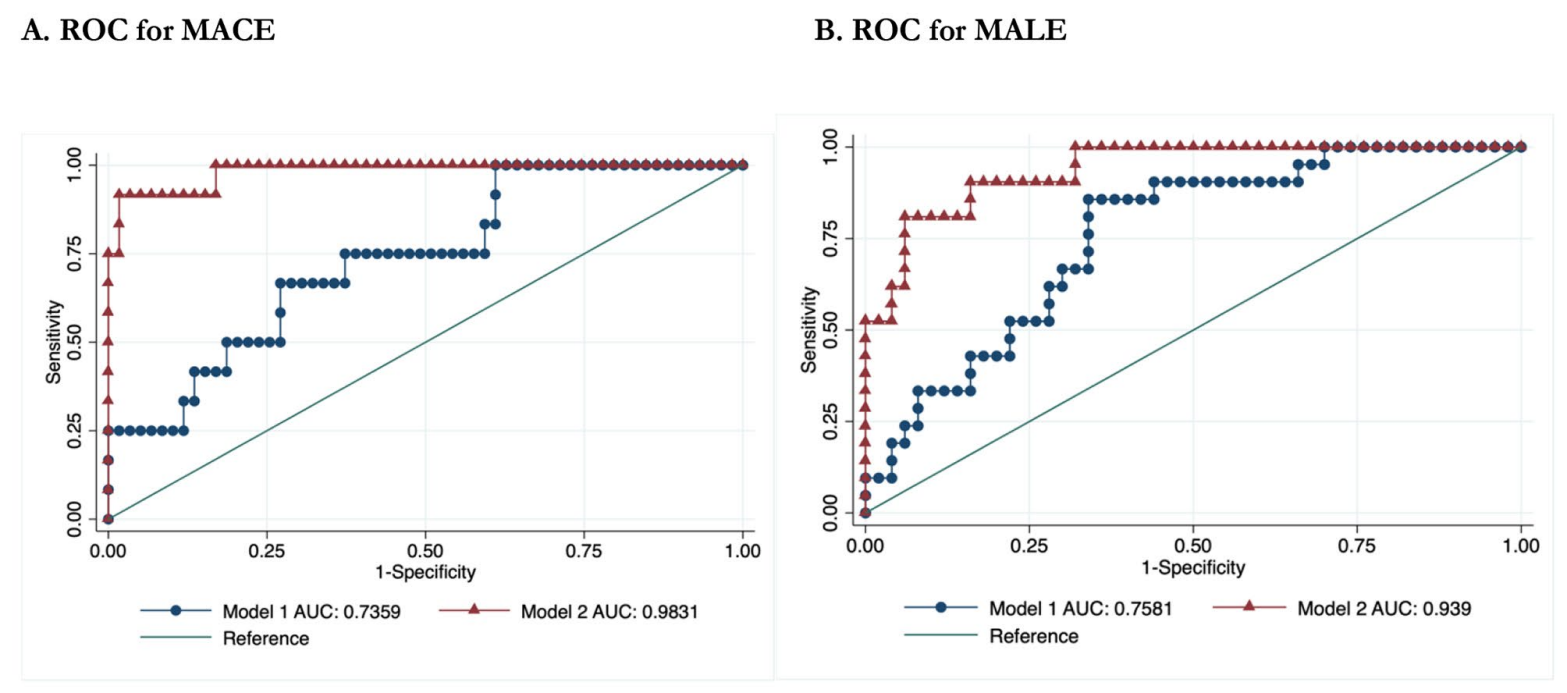
A. ROC for MACE B. ROC for MALE Receiver operating characteristic (ROC) curves comparing the performance of a model without (Model 1) and with (Model 2) biomarkers in predicting MACE (**A**) and MALE (**B**). The true-positive rate (sensitivity) is plotted as a function of the false-positive rate (1 - Specificity). p < 0.01

The baseline ROC model included age, sex, BMI, high blood pressure, diabetes duration, smoking status, Rutherford staging, previous cardiovascular and cerebrovascular events, treatment, total cholesterol, LDL-C, triglycerides, FBG, and HbA1c. The comparison of ROC curves between the baseline model with only clinical and laboratory risk factors and the model including our panel of biomarkers is reported in Fig. 3A for MACE and Fig. 3B for MALE. In both cases, including the biomarker panel significantly improved the prediction of incident events: for MACE, the area under the ROC curve was 0.73 [95% confidence interval (CI) 0.67, 0.79] for the baseline model and 0.98 (95% CI 0.95, 0.99, p < 0.01) for the model with cytokines. For MALE, the area under the ROC curve was 0.76 (95% CI 0.71, 0.81) for the baseline model and 0.94 (95% CI 0.91, 0.98, p < 0.01) for the model with cytokines.

## Discussion

Diabetic patients with CLTI undergoing LER have a very high risk of MACE and MALE in the 12-month period following treatment [7]. Best medical therapies and risk factors control do not prevent the development of such complications [2]. In clinical practice, during the post-revascularization period patients with similar clinical features and endovascular approaches may unpredictably experience completely different procedural outcomes. Therefore, there is an urgent need to develop and validate non-invasive tools that include traditional and non-traditional risk factors for the prediction of the occurrence of complications after LER in this particular population.

It is well established that inflammation plays a key role in the complications of diabetes mellitus [39]. Several studies have shown that the pre-procedural inflammatory state is related to an increased risk for restenosis after coronary angioplasty [40]. We hypothesized that patients with an activation of the systemic inflammatory cascade and reduction of endogenous anti-inflammatory mechanisms have worse outcomes. The results of this study show that the baseline levels of IL-1, IL-6, TNF-α, CRP, HMGB-1, OPG and Sortilin were increased in patients who developed MALE and MACE during follow-up compared to those who did not present these outcomes. These findings are in agreement with previous studies that have individually analyzed each biomarker [7, 18, 23, 28]. Instead, Omentin-1 serum levels were reduced in patients who presented MALE and MACE, compared to patients who had no events. This inverse correlation has been previously found in populations affected by CAD [41], PAD [30, 31] and carotid artery stenosis [42] and confirms the anti-inflammatory role of this adipokine [43–46]. Remarkably, it is the first time this panel of cytokines has been studied simultaneously in the same population of patients.

In recent years, the CANTOS study raised the attention on the role of IL-1 in cardiovascular diseases. Researchers have shown that the intake of canakinumab, a monoclonal antibody directed against IL-1, that is used in clinical practice for treatment of some rheumatological diseases, significantly reduced the risk of recurrence of cardiovascular events in patients with history of myocardial infarction and persistently high CRP levels, confirming the close link between inflammation and atherosclerosis [14]. To our knowledge, this is the first prospective study that demonstrates the association of IL-1 and cardiovascular and limbs events in diabetic patients after endovascular revascularization for CLTI.

The multivariate analysis for MACE showed that, after adjusting for the factors of conventional cardiovascular risk and all biomarkers in analysis, IL-6, OPG e CRP levels are independently associated with the outcome. The ROC curve confirmed their high predictive power. These results confirm the association between IL-6, OPG, CRP and cardiovascular outcomes that already emerged in one of our previous study [7]. Multivariate analysis for MALE showed that after adjusting for factors of conventional cardiovascular risk and all cytokines in analysis, CRP, HMGB-1, OPG and Omentin-1 are independently associated with the outcome. The ROC curve confirmed their high predictive power. These results confirm the correlation between CRP, HMGB-1, OPG and Omentin-1 already highlighted in previous studies [7, 18, 23, 31].

In the past years, the little of awareness in PAD has led to scarce tools for the early diagnosis, progression and prognosis evaluation, resulting in inadequate therapeutic interventions. Non-invasive circulating serum biomarkers may be predictive effective tools in this setting. Several studies have evaluated the possible role of different molecules in the diagnosis and prognosis of PAD [47]. These studies have also offered insights into the complex pathophysiological mechanisms and numerous molecular pathways that are involved in PAD. Therefore, assessment of a single biomarker may not reflect those complex interactions [47]. Instead, the combination of a panel of biomarkers and conventional cardiovascular risk factors could be useful to obtain more accurate prediction algorithms.

There is a scarcity of scientific research on predictive models for MACE and MALE after LER in diabetic patients with CLTI. In a retrospective study, Stone et al. found that, after LER, elevated pre-procedural CRP levels were associated with MALE, and elevated levels of CRP and brain natriuretic peptide (BNP) were associated with late cardiovascular events [48]. Berger et al. identified 11 predictors, including patient age over 75 years, history of PAD, dementia, diabetes, hypertension, renal disease, cerebrovascular disease, chronic heart failure, smoking status, prior myocardial infarction, and chronic pulmonary disease, that are associated with the incidence of MACE/MALE in CAD and/or PAD patients [49]. Vieceli Dalla Sega et al. measured the circulating levels of a panel of 23 molecules related to inflammation, endothelial dysfunction, platelet activation, and thrombophilia in 92 patients with CLTI and diabetic foot ulcers requiring PTA and foot surgery. They found that PAI-1 and endothelin-1 are associated with the need for new revascularization and, the levels of thrombomodulin and sCD40L are associated with new lesions or recurrence [50]. Some traditional risk factors are negatively associated with MACE and MALE in our population. As far as Rutherford staging is concerned, the predictive efficacy of the Rutherford classification in determining post-revascularization outcomes has not been adequately examined. While it may be intuitive to assume that a higher Rutherford grade indicates a higher risk of failure, it is important to consider that the presence of infection at baseline might have a stronger influence on risk stratification, as previous research has suggested [51]. While no formal protocol was used to rule out infections, patients with active or recent infections were excluded in this study. Furthermore, it is conceivable that patients who had more advanced stages at baseline underwent more careful management of the lesions and therefore experienced fewer MALE. As regards previous smoking habit, the definition of no smokers in this study was attributed to those who had never smoked in the past, while the definition of the National Center for Health Statistics defines as a non-smoker “An adult who has never smoked, or who has smoked less than 100 cigarettes in his or her lifetime”. Additionally, we were unable to ascertain the pack-years of smoking for the population of current and former smokers. Thus, It is also conceivable that the population of former smokers had ceased smoking for an extended period, thereby decreasing the cardiovascular risk. This could justify, at least in part, the negative association with MACE and the search for more objective and measurable biomarkers.

To our knowledge, the present study is the first to demonstrate that a panel of circulating biomarkers easily measurable could predict the incidence of MACE and MALE over 90% in diabetic patients with CLTI after LER.

### Study limitations

A limitation of this single-center study is the small number of patients included. However, this is a pilot study conducted on a carefully selected population with restrictive characteristics in order to select a population as homogeneous as possible. Based on the limited sample size analyzed, it was not possible to perform a comprehensive analysis of each individual biomarker in relation to individual conventional risk factors. A further limitation of the study is the lack of a formal protocol to rule out possible bone infections (for example using standard radiographic examinations or computed tomography or magnetic resonance imaging of bone), although patients with obvious signs of infections were excluded from recruitment. The aim of this research was to find easily accessible and reproducible biomarkers for the typical diabetic patient with CLTI. The study also did not clarify the effect that the revascularization procedure could have on the levels of analyzed biomarkers. Therefore, it might be helpful to monitor cytokine levels after the procedure and in the follow-up period in order to evaluate the effect of the variations of these on MACE and MALE occurrence. In addition, more conclusive evidence about the real causal relationship between the studied biomarkers and the outcomes after LER in diabetic patients will only be found after clinical multi-centric studies, on larger cohorts and longer follow-up periods. Finally, our data doesn’t offer results that can immediately change the clinical management of patients. However, this type of research is essential to fill the gap that the classic biomarkers (e.g., glycated hemoglobin, LDL-cholesterol) cannot resolve, regarding the chance of predicting the development of MACE and MALE. It is well-established that diabetic patients with PAD and similar risk factors can experience unpredictable vascular outcomes, indicating the presence of unknown or uncontrollable, at least at the moment, risk factors[52]. Currently, the residual cardiovascular risk in our patients cannot be precisely measured, but it is clear that it exceeds 50% despite the best therapies available for diabetes, hypercholesterolemia, and hypertension[53]. In this scenario, while CRP is a nonspecific biomarker, it holds potential in stratifying residual inflammatory risk and identifying a subgroup of patients who might benefit from additional anti-inflammatory treatments, like colchicine or canakinumab[54]. Additionally, considering the close association between Sortilin and PCSK9[55], the systematic evaluation of Sortilin as a biomarker could help identify patients who may benefit from early initiation of therapy with PCSK9 inhibitors. Other biomarkers under investigation may also contribute to both diagnosis and treatment. However, it is important to acknowledge that relying on a single biomarker alone may be insufficient. Utilizing a panel of biomarkers could offer a more comprehensive assessment, enabling a more tailored approach to individual patient profiles.

## Conclusion

Our study showed that elevated levels of IL-1, IL-6, TNF-α, PCR, HMGB-1, OPG and Sortilin and reduced Omentin-1 levels correlate with worse vascular outcomes in CLTI diabetic patients after LER. Hence, they hold significant potential as biomarkers, given their ease of measurement and reproducibility. These parameters can reliably predict the likelihood of complications that a treated patient may encounter. A standardized score that combines conventional cardiovascular risk factors and these biomarkers could enable clinicians to assess patients’ risk profile, identify patients most susceptible to unfavorable outcomes and choose the most appropriate and personalized therapeutic and follow-up approach. While these findings require validation on a larger patient cohort, they indicate progress in the management of this complex and disabling condition.

## Electronic supplementary material

Below is the link to the electronic supplementary material.

Supplementary Material 1

## Data Availability

The datasets generated during the current study are available from the corresponding author on reasonable request.

## Declarations

### Guarantor’s statement

Dr. Andrea Flex is the guarantor of this work and, as such, had full access to all the data in the study and takes responsibility for the integrity of the data and the accuracy of the data analysis.

## List of Abbreviations

BMI: Body mass index
CAD: Coronary artery disease
CLTI: Chronic limb-threatening ischemia
CRP: C-Reactive Protein
CVD: Cerebrovascular disease
eGFR: Estimated glomerular filtration rate
FBG: Fasting blood glucose
HbA1c: Glycated hemoglobin
HMGB-1: High-Mobility Group Box-1
IL-1: Interleukin-1
IL-6: Interleukin-6
LDL-C: Low-density lipoprotein cholesterol
LER: Lower limb revascularization
MACE: Major adverse cardiovascular events
MALE: Major adverse limb events
OPG: Osteoprotegerin
PAD: Peripheral artery disease
ROC: Receiver operating characteristics
T2DM: Type 2 diabetes mellitus
TNF-α: Tumor Necrosis Factor-α

## References

[1] Vrsalovic M (2018). Diabetes and peripheral artery disease: a bad combination. Am J Surg.

[2] Conte MS, Bradbury AW, Kolh P, White JV, Dick F, Fitridge R, Mills JL, Ricco JB, Suresh KR, Murad MH (2019). Global vascular guidelines on the management of chronic limb-threatening ischemia. Eur J Vasc Endovasc Surg.

[3] Stavroulakis K, Borowski M, Torsello G, Bisdas T, Collaborators C (2018). One-year results of first-line treatment strategies in patients with critical limb ischemia (CRITISCH Registry). J Endovasc Ther.

[4] Torsello G, Stavroulakis K, Brodmann M, Micari A, Tepe G, Veroux P, Benko A, Choi D, Vermassen FEG, Jaff MR et al. Three-year sustained clinical efficacy of drug-coated balloon angioplasty in a real-world femoropopliteal cohort. J Endovasc Ther 2020:1526602820931477. 10.1177/1526602820931477 PMC7545651 32583749

[5] Farber A, Menard MT, Conte MS, Kaufman JA, Powell RJ, Choudhry NK, Hamza TH, Assmann SF, Creager MA, Cziraky MJ (2022). Surgery or endovascular therapy for chronic limb-threatening ischemia. N Engl J Med.

[6] Biscetti F, Nardella E, Rando MM, Cecchini AL, Gasbarrini A, Massetti M, Flex A. Outcomes of Lower Extremity Endovascular Revascularization: potential predictors and Prevention Strategies. Int J Mol Sci 2021, 22(4). 10.3390/ijms22042002 PMC7922574 33670461

[7] Biscetti F, Ferraro PM, Hiatt WR, Angelini F, Nardella E, Cecchini AL, Santoliquido A, Pitocco D, Landolfi R, Flex A. Inflammatory Cytokines Associated with failure of Lower Extremity Endovascular Revascularization (LER): a prospective study of a Population with Diabetes. Diabetes Care 2019. 10.2337/dc19-0408 31371431

[8] Anand SS, Caron F, Eikelboom JW, Bosch J, Dyal L, Aboyans V, Abola MT, Branch KRH, Keltai K, Bhatt DL (2018). Major adverse limb events and mortality in patients with peripheral artery disease: the COMPASS trial. J Am Coll Cardiol.

[9] Hinchliffe RJ, Brownrigg JR, Andros G, Apelqvist J, Boyko EJ, Fitridge R, Mills JL, Reekers J, Shearman CP, Zierler RE (2016). Effectiveness of revascularization of the ulcerated foot in patients with diabetes and peripheral artery disease: a systematic review. Diab/Metab Res Rev.

[10] Cesari M, Penninx BW, Newman AB, Kritchevsky SB, Nicklas BJ, Sutton-Tyrrell K, Rubin SM, Ding J, Simonsick EM, Harris TB (2003). Inflammatory markers and onset of cardiovascular events: results from the Health ABC study. Circulation.

[11] Burger-Kentischer A, Goebel H, Seiler R, Fraedrich G, Schaefer HE, Dimmeler S, Kleemann R, Bernhagen J, Ihling C (2002). Expression of macrophage migration inhibitory factor in different stages of human atherosclerosis. Circulation.

[12] Biscetti F, Straface G, Bertoletti G, Vincenzoni C, Snider F, Arena V, Landolfi R, Flex A (2015). Identification of a potential proinflammatory genetic profile influencing carotid plaque vulnerability. J Vasc Surg.

[13] Vromman A, Ruvkun V, Shvartz E, Wojtkiewicz G, Santos Masson G, Tesmenitsky Y, Folco E, Gram H, Nahrendorf M, Swirski FK (2019). Stage-dependent differential effects of interleukin-1 isoforms on experimental atherosclerosis. Eur Heart J.

[14] Ridker PM, Everett BM, Thuren T, MacFadyen JG, Chang WH, Ballantyne C, Fonseca F, Nicolau J, Koenig W, Anker SD (2017). Antiinflammatory therapy with Canakinumab for atherosclerotic disease. N Engl J Med.

[15] Biscetti F, Straface G, De Cristofaro R, Lancellotti S, Rizzo P, Arena V, Stigliano E, Pecorini G, Egashira K, De Angelis G (2010). High-mobility group box-1 protein promotes angiogenesis after peripheral ischemia in diabetic mice through a VEGF-dependent mechanism. Diabetes.

[16] Flex A, Gaetani E, Angelini F, Sabusco A, Chillà C, Straface G, Biscetti F, Pola P, Castellot JJ, Pola R (2007). Pro-inflammatory genetic profiles in subjects with peripheral arterial occlusive disease and critical limb ischemia. J Intern Med.

[17] Biscetti F, Flex A, Alivernini S, Tolusso B, Gremese E, Ferraccioli G (2017). The role of high-mobility Group Box-1 and its crosstalk with Microbiome in Rheumatoid Arthritis. Mediators Inflamm.

[18] Giovannini S, Tinelli G, Biscetti F, Straface G, Angelini F, Pitocco D, Mucci L, Landolfi R, Flex A (2017). Serum high mobility group box-1 and osteoprotegerin levels are associated with peripheral arterial disease and critical limb ischemia in type 2 diabetic subjects. Cardiovasc Diabetol.

[19] Biscetti F, Porreca CF, Bertucci F, Straface G, Santoliquido A, Tondi P, Angelini F, Pitocco D, Santoro L, Gasbarrini A (2014). TNFRSF11B gene polymorphisms increased risk of peripheral arterial occlusive disease and critical limb ischemia in patients with type 2 diabetes. Acta Diabetol.

[20] Biscetti F, Pitocco D, Straface G, Zaccardi F, de Cristofaro R, Rizzo P, Lancellotti S, Arena V, Stigliano E, Musella T (2011). Glycaemic variability affects ischaemia-induced angiogenesis in diabetic mice. Clin Sci (Lond).

[21] Biscetti F, Ghirlanda G, Flex A (2011). Therapeutic potential of high mobility group box-1 in ischemic injury and tissue regeneration. Curr Vasc Pharmacol.

[22] Biscetti F, Rando MM, Nardella E, Cecchini AL, Pecorini G, Landolfi R, Flex A. High mobility Group Box-1 and Diabetes Mellitus Complications: state of the art and future perspectives. In: Int J Mol Sci vol. 20; 2019. 10.3390/ijms20246258 PMC6940913 31835864

[23] Rando MM, Biscetti F, Cecchini AL, Nardella E, Nicolazzi MA, Angelini F, Iezzi R, Eraso LH, Dimuzio PJ, Pitocco D (2022). Serum high mobility group box-1 levels associated with cardiovascular events after lower extremity revascularization: a prospective study of a diabetic population. Cardiovasc Diabetol.

[24] Schoppet M, Preissner KT, Hofbauer LC (2002). RANK ligand and osteoprotegerin: paracrine regulators of bone metabolism and vascular function. Arterioscler Thromb Vasc Biol.

[25] Straface G, Biscetti F, Pitocco D, Bertoletti G, Misuraca M, Vincenzoni C, Snider F, Arena V, Stigliano E, Angelini F (2011). Assessment of the genetic effects of polymorphisms in the osteoprotegerin gene, TNFRSF11B, on serum osteoprotegerin levels and carotid plaque vulnerability. Stroke.

[26] Oh TJ, Ahn CH, Kim BR, Kim KM, Moon JH, Lim S, Park KS, Lim C, Jang H, Choi SH (2017). Circulating sortilin level as a potential biomarker for coronary atherosclerosis and diabetes mellitus. Cardiovasc Diabetol.

[27] Biscetti F, Bonadia N, Santini F, Angelini F, Nardella E, Pitocco D, Santoliquido A, Filipponi M, Landolfi R, Flex A (2019). Sortilin levels are associated with peripheral arterial disease in type 2 diabetic subjects. Cardiovasc Diabetol.

[28] Biscetti F, Nardella E, Rando MM, Cecchini AL, Bonadia N, Bruno P, Angelini F, Di Stasi C, Contegiacomo A, Santoliquido A (2020). Sortilin levels correlate with major cardiovascular events of diabetic patients with peripheral artery disease following revascularization: a prospective study. Cardiovasc Diabetol.

[29] De Jager SC, Pasterkamp G (2016). Atheroprotective properties of human Omentin-1 in experimental atherosclerosis. Cardiovascular Res.

[30] Biscetti F, Nardella E, Bonadia N, Angelini F, Pitocco D, Santoliquido A, Filipponi M, Landolfi R, Flex A (2019). Association between plasma omentin-1 levels in type 2 diabetic patients and peripheral artery disease. Cardiovasc Diabetol.

[31] Biscetti F, Nardella E, Rando MM, Cecchini AL, Angelini F, Cina A, Iezzi R, Filipponi M, Santoliquido A, Pitocco D (2020). Association between omentin-1 and major cardiovascular events after lower extremity endovascular revascularization in diabetic patients: a prospective cohort study. Cardiovasc Diabetol.

[32] Rutherford RB, Baker JD, Ernst C, Johnston KW, Porter JM, Ahn S, Jones DN (1997). Recommended standards for reports dealing with lower extremity ischemia: revised version. J Vasc Surg.

[33] Gerhard-Herman MD, Gornik HL, Barrett C, Barshes NR, Corriere MA, Drachman DE, Fleisher LA, Fowkes FG, Hamburg NM, Kinlay S (2017). 2016 AHA/ACC Guideline on the management of patients with lower extremity peripheral artery disease: a report of the American College of Cardiology/American Heart Association Task Force on Clinical Practice Guidelines. Circulation.

[34] Thukkani AK, Kinlay S (2015). Endovascular intervention for peripheral artery disease. Circ Res.

[35] Sacks D, Marinelli DL, Martin LG, Spies JB, Committee SoIRTA (2003). Reporting standards for clinical evaluation of new peripheral arterial revascularization devices. J Vasc Interv Radiol.

[36] Eikelboom JW, Connolly SJ, Bosch J, Dagenais GR, Hart RG, Shestakovska O, Diaz R, Alings M, Lonn EM, Anand SS (2017). Rivaroxaban with or without aspirin in stable Cardiovascular Disease. N Engl J Med.

[37] Stevens LA, Schmid CH, Greene T, Zhang YL, Beck GJ, Froissart M, Hamm LL, Lewis JB, Mauer M, Navis GJ (2010). Comparative performance of the CKD epidemiology collaboration (CKD-EPI) and the modification of Diet in Renal Disease (MDRD) Study equations for estimating GFR levels above 60 mL/min/1.73 m2. Am J Kidney Dis.

[38] Aboyans V, Ricco JB, Bartelink MEL, Björck M, Brodmann M, Cohnert T, Collet JP, Czerny M, De Carlo M, Debus S et al. 2017 ESC Guidelines on the diagnosis and treatment of peripheral arterial Diseases, in collaboration with the european society for vascular surgery (ESVS): document covering atherosclerotic disease of extracranial carotid and vertebral, mesenteric, renal, upper and lower extremity arteriesEndorsed by: the european Stroke Organization (ESO)the Task Force for the diagnosis and treatment of peripheral arterial Diseases of the European Society of Cardiology (ESC) and of the european society for vascular surgery (ESVS). Eur Heart J 2017.

[39] Signorelli SS, Katsiki N (2018). Oxidative stress and inflammation: their role in the pathogenesis of Peripheral Artery Disease with or without type 2 diabetes Mellitus. Curr Vasc Pharmacol.

[40] Schillinger M, Minar E (2005). Restenosis after percutaneous angioplasty: the role of vascular inflammation. Vasc Health Risk Manag.

[41] Du Y, Ji Q, Cai L, Huang F, Lai Y, Liu Y, Yu J, Han B, Zhu E, Zhang J (2016). Association between omentin-1 expression in human epicardial adipose tissue and coronary atherosclerosis. Cardiovasc Diabetol.

[42] Nishimura M, Morioka T, Hayashi M, Kakutani Y, Yamazaki Y, Kurajoh M, Mori K, Fukumoto S, Shioi A, Shoji T (2019). Plasma omentin levels are inversely associated with atherosclerosis in type 2 diabetes patients with increased plasma adiponectin levels: a cross-sectional study. Cardiovasc Diabetol.

[43] Yamawaki H (2011). Vascular effects of novel adipocytokines: focus on vascular contractility and inflammatory responses. Biol Pharm Bull.

[44] Yoo HJ, Hwang SY, Hong HC, Choi HY, Yang SJ, Seo JA, Kim SG, Kim NH, Choi KM, Choi DS (2011). Association of circulating omentin-1 level with arterial stiffness and carotid plaque in type 2 diabetes. Cardiovasc Diabetol.

[45] Watanabe K, Watanabe R, Konii H, Shirai R, Sato K, Matsuyama TA, Ishibashi-Ueda H, Koba S, Kobayashi Y, Hirano T (2016). Counteractive effects of omentin-1 against atherogenesis†. Cardiovascular Res.

[46] Hiramatsu-Ito M, Shibata R, Ohashi K, Uemura Y, Kanemura N, Kambara T, Enomoto T, Yuasa D, Matsuo K, Ito M (2016). Omentin attenuates atherosclerotic lesion formation in apolipoprotein E-deficient mice. Cardiovascular Res.

[47] Saenz-Pipaon G, Martinez-Aguilar E, Orbe J, González Miqueo A, Fernandez-Alonso L, Paramo JA, Roncal C. The role of circulating biomarkers in peripheral arterial disease. Int J Mol Sci 2021, 22(7). 10.3390/ijms22073601 PMC8036489 33808453

[48] Stone PA, Schlarb H, Campbell JE, Williams D, Thompson SN, John M, Campbell JR, AbuRahma AF (2014). C-reactive protein and brain natriuretic peptide as predictors of adverse events after lower extremity endovascular revascularization. J Vasc Surg.

[49] Berger A, Simpson A, Leeper NJ, Murphy B, Nordstrom B, Ting W, Zhao Q, Berger J (2020). Real-world predictors of major adverse Cardiovascular events and major adverse limb events among patients with chronic coronary artery disease and/or peripheral arterial disease. Adv Ther.

[50] Vieceli Dalla Sega F, Cimaglia P, Manfrini M, Fortini F, Marracino L, Bernucci D, Pompei G, Scala A, Trichilo M, De Carolis B et al. Circulating biomarkers of endothelial dysfunction and inflammation in Predicting Clinical Outcomes in Diabetic patients with critical limb ischemia. Int J Mol Sci 2022, 23(18). 10.3390/ijms231810641 PMC9506462 36142551

[51] Abualhin M, Gargiulo M, Bianchini Massoni C, Mauro R, Morselli-Labate AM, Freyrie A, Faggioli G, Stella A (2019). A prognostic score for clinical success after revascularization of critical limb ischemia in hemodialysis patients. J Vasc Surg.

[52] Aday AW, Matsushita K (2021). Epidemiology of Peripheral Artery Disease and Polyvascular Disease. Circ Res.

[53] Hernandez JL, Lozano FS, Riambau V, Almendro-Delia M, Cosin-Sales J, Bellmunt-Montoya S, Garcia-Alegria J, Garcia-Moll X, Gomez-Doblas JJ, Gonzalez-Juanatey JR et al. Reducing residual thrombotic risk in patients with peripheral artery disease: impact of the COMPASS trial. Drugs Context 2020, 9. 10.7573/dic.2020-5-5 PMC7357685 32699549

[54] Ajala ON, Everett BM (2020). Targeting inflammation to reduce residual Cardiovascular risk. Curr Atheroscler Rep.

[55] Hu D, Yang Y, Peng DQ (2017). Increased sortilin and its independent effect on circulating proprotein convertase subtilisin/kexin type 9 (PCSK9) in statin-naive patients with coronary artery disease. Int J Cardiol.

