# Supplementary material for: Development of a biomarker panel for assessing cardiovascular risk in diabetic patients with chronic limb-threatening ischemia (CLTI): a prospective study
Source: Cardiovasc Diabetol. 2023 Jun 12;22:136. doi: 10.1186/s12933-023-01872-x (PMC10262545; doi:10.1186/s12933-023-01872-x)
Supplement: Supplementary file 1 — Supplementary Material 1 [file 12933_2023_1872_MOESM1_ESM.docx]

**Supplementary Tables**

**Supplementary Table 1. Demographic and clinical data of study participants without or with MACE**

|  | **NO MACE (n = 222)** | **MACE (n = 42)** | **p value** |
| --- | --- | --- | --- |
| Men/female, n | 150:72 | 21:21 | 0.03 |
| Age, years ± SD | 72.8 ± 8.8 | 76.5 ± 8.3 | 0.13 |
| Diabetes duration, years ± SD | 14.4 ± 12.9 | 25.6 ± 14.2 | <0.01 |
| BMI, Kg/m^2^ ± SD | 25.8 ± 4.4 | 29.9 ± 6.4 | <0.01 |
| Smoking (current), n (%) | 57 (25.6) | 6 (14.3) | 0.11 |
| Smoking (former), n (%) | 102 (45.9) | 18 (42.8) | 0.71 |
| Hypertension, n (%) | 174 (78.3) | 30 (71.4) | 0.32 |
| Hypercholesterolemia, n (%) | 204 (91.9) | 39 (92.8) | 0.04 |
| CAD, n (%) | 81 (36.5) | 36 (85.7) | <0.01 |
| CVD, n (%) | 78 (35.1) | 21 (50.0) | 0.07 |
| Rutherford II-4, n (%) | 54 (24.3) | 0 (0.0) | <0.01 |
| Rutherford III-5, n (%) | 84 (37.8) | 24 (57.1) | 0.20 |
| Rutherford III-6, n (%) | 30 (13.5) | 12 (28.6) | 0.01 |
| HbA1c, % ± SD | 7.1 ± 1.1 | 6.9 ± 1.2 | 0.39 |
| FBG, mg/dL ± SD | 130.8 ± 52.8 | 143.2 ± 62.3 | 0.18 |
| Total cholesterol, mg/dL ± SD | 136.3 ± 35.8 | 123.9 ± 26.4 | 0.48 |
| LDL cholesterol, mg/dL ± SD | 72.4 ± 29.6 | 65.4 ± 20.2 | 0.18 |
| HDL cholesterol, mg/dL ± SD | 38.8 ± 12.3 | 35.4 ± 8.7 | 0.11 |
| Triglycerides, mg/dL ± SD | 120.7 ± 67.4 | 111.2 ± 32.8 | 0.39 |
| Creatinine, mg/dL ± SD | 1.6 ± 1.7 | 2.7 ± 2.2 | <0.01 |

**Supplementary Table 2. Demographic and clinical data of study participants without or with MALE**

|  | **NO MALE (n = 183)** | **MALE (n = 81)** | **p value** |
| --- | --- | --- | --- |
| Men/female, n | 111:72 | 60:21 | 0.03 |
| Age, years ± SD | 74.8 ± 8.5 | 70.2 ± 8.7 | <0.01 |
| Diabetes duration, years ± SD | 16.0 ± 13.6 | 16.8 ± 14.1 | 0.67 |
| BMI, Kg/m^2^ ± SD | 26.15 ± 4.9 | 27.1 ± 5.2 | 0.14 |
| Smoking (current), n (%) | 45 (24.6) | 18 (22.2) | 0.68 |
| Smoking (former), n (%) | 75 (41.0) | 45 (55.6) | 0.28 |
| Hypertension, n (%) | 144 (78.7) | 60 (74.1) | 0.41 |
| Hypercholesterolemia, n (%) | 162 (88.5) | 81 (100.0) | <0.01 |
| CAD, n (%) | 69 (37.7) | 48 (59.2) | <0.01 |
| CVD, n (%) | 60 (32.8) | 39 (48.1) | 0.02 |
| Rutherford II-4, n (%) | 45 (24.6) | 9 (11.1) | 0.01 |
| Rutherford III-5, n (%) | 75 (41.0) | 33 (40.7) | 0.97 |
| Rutherford III-6, n (%) | 27 (14.7) | 15 (18.5) | 0.44 |
| HbA1c, % ± SD | 7.0 ± 1.1 | 7.3 ± 1.3 | 0.13 |
| FBG, mg/dL ± SD | 130.8 ± 57.0 | 128.2 ± 48.4 | 0.37 |
| Total cholesterol, mg/dL ± SD | 137.3 ± 34.0 | 128.3 ± 35.9 | 0.06 |
| LDL cholesterol, mg/dL ± SD | 72.9 ± 28.2 | 68.1 ± 29.2 | 0.20 |
| HDL cholesterol, mg/dL ± SD | 39.9 ± 12.2 | 34.9 ± 10.5 | <0.01 |
| Triglycerides, mg/dL ± SD | 120.4 ± 71.7 | 116.7 ± 40.0 | 0.65 |
| Creatinine, mg/dL ± SD | 1.7 ± 1.9 | 1.8 ± 1.7 | 0.59 |
